# EGFR signaling suppresses type 1 cytokine-induced T-cell attracting chemokine secretion in head and neck cancer

**DOI:** 10.1371/journal.pone.0203402

**Published:** 2018-09-07

**Authors:** Wenbo Ma, Fernando Concha-Benavente, Saskia J. A. M. Santegoets, Marij J. P. Welters, Ilina Ehsan, Robert L. Ferris, Sjoerd H. van der Burg

**Affiliations:** 1 Department of Medical Oncology, Leiden University Medical Center, Leiden, The Netherlands; 2 Department of Otolaryngology, University of Pittsburgh, and the University of Pittsburgh Cancer Institute, Pittsburgh, PA, United States of America; Marshall University, UNITED STATES

## Abstract

Resistance to antitumor immunity can be promoted by the oncogenic pathways operational in human cancers, including the epidermal growth factor receptor (EGFR) pathway. Here we studied if and how EGFR downstream signaling in head and neck squamous cell carcinoma (HNSCC) can affect the attraction of immune cells. HPV-negative and HPV-positive HNSCC cell lines were analyzed in vitro for CCL2, CCL5, CXCL9, CXCL10, IL-6 and IL-1β expression and the attraction of T cells under different conditions, including cetuximab treatment and stimulation with IFNγ and TNFα using qPCR, ELISA and migration experiments. Biochemical analyses with chemical inhibitors and siRNA transfection were used to pinpoint the underlying mechanisms. Stimulation of HNSCC cells with IFNγ and TNFα triggered the production of T-cell attracting chemokines and required c-RAF activation. Blocking of the EGFR with cetuximab during this stimulation increased chemokine production in vitro, and augmented the attraction of T cells. Mechanistically, cetuximab decreased the phosphorylation of MEK1, ERK1/2, AKT, mTOR, JNK, p38 and ERK5. Chemical inhibition of EGFR signaling showed a consistent and pronounced chemokine production with MEK1/2 inhibitor PD98059 and JNK inhibitor SP600125, but not with inhibitors of p38, PI3K or mTOR. Combination treatment with cetuximab and a MEK1/2 or JNK inhibitor induced the highest chemokine expression. In conclusion, overexpression of EGFR results in the activation of multiple downstream signaling pathways that act simultaneously to suppress type 1 cytokine stimulated production of chemokines required to amplify the attraction of T cells.

## Introduction

Head and neck squamous cell carcinoma (HNSCC) is the sixth most common malignancy worldwide. The risk factors of HNSCC are tobacco and alcohol use or an infection with high-risk human papillomaviruses, in particular type 16 (HPV16) [1]. Previous studies have shown that patients with strongly T-cell infiltrated HNSCC display better survival [2–4]. Interestingly, the group of patients with an HPV-induced HNSCC have a much better prognosis than their HPV-negative counterparts [5] and this was related to heavy tumor-infiltration by activated CD4+ and CD8+ T cells [6–8]. Importantly, a prospective study on the role of tumor-specific T-cell responses in HNSCC showed that the viral antigens in HPV16+ HNSCC triggered an intratumoral IFNγ- and TNFα-producing HPV-specific T cell response which shaped a favorable type 1 immune contexture and was strongly associated with a good clinical response to standard (chemo)radiotherapy [9]. HNSCC patients that are refractory to first line therapy may respond upon treatment with an anti-programmed cell death protein-1(PD-1) monoclonal antibody [10]. Together, these data reveal that the immune response play an important role in HNSCC but also that in many cases such a response is lacking.

The epidermal growth factor receptor (EGFR) is frequently overexpressed in cancers of patients with poor prognosis and is found in 80–90% of HNSCC [11]. EGFR overexpression results in increased cell proliferation, cell migration and resistance to apoptosis. Based on this a number of agents targeting EGFR have been developed and are now used to treat HNSCC patients, one of which is the monoclonal antibody cetuximab [12]. Cetuximab blockade of EGFR will inhibit tumor growth, DNA damage repair, and metastasis. It does so by interfering with the binding of the natural ligands to EGFR as well as by inducing receptor endocytosis, thereby disrupting EGFR signaling. Furthermore, cetuximab may also trigger antibody dependent cell-mediated cytotoxicity [13] More recent data, however, suggest that activation of EGFR signaling itself also bears an immune regulatory component. EGFR activation represses MHC class I and II expression [14] as well as promotes the expression of PD-L1 in lung cancers and HNSCC [15, 16]. Furthermore, the presence of an EGFR mutation is related to lower T-cell infiltration [17]. The downstream targets of EGFR include the Raf-MEK-ERK pathway, the MAP3K pathway and the PI3K-AKT-mTOR pathway [18]. Interestingly, loss of PTEN resulted in decreased T-cell infiltration and resistance to PD-1 blocking in preclinical models of melanoma. T cell infiltration and tumor control could be restored by blocking PI3K-AKT signaling using the inhibitor PI3Kβ [19]. These data suggest that the EGFR downstream signaling pathways may, similar to other recently reported oncogenic pathways [20, 21], attenuate tumor immunity by preventing T cell infiltration.

In order to study if and how EGFR downstream signaling may affect immune infiltration, we made use of HPV- and HPV+ HNSCC cell lines which were stimulated with type 1 cytokines in the absence and presence of cetuximab or inhibitors of molecules downstream of the EGFR. We show that blocking EGFR with cetuximab inhibits the activation of several pathways downstream of EGFR and results in an increased production of inflammatory chemokines and attraction of T cells when the tumor cells are stimulated with IFNγ and TNFα. Mechanistically, EGFR signaling suppressed type 1 cytokine-induced chemokine production in a MEK and JNK dependent fashion.

## Material and methods

### Cell culture

The HNSCC cell lines were obtained from the University of Michigan (Ann Arbor, MI, USA) and designated as UM-SCC. We obtained UM-SCC4 and UM-SCC19 (both HPV negative) as well as UM-SCC47 and UM-SCC104 (both HPV16-positive) in 2012. The cells were cultured in RPMI 1640 (Gibco/ Thermo Fisher Scientific (TFS) Bleiswijk, the Netherlands) with 10% Fetal Calf Serum (PAA laboratories; Pashing, Austria) and penicillin/streptomycin (TFS). Microsatellite analysis was performed in July 2016 by BaseClear (Leiden, the Netherlands) to assure cell line authentication when the experiments were performed. Mycoplasma was tested on a monthly basis and found negative.

### Patients and specimens

All patients signed an informed consent approved by the Institutional Review Board (IRB #99–06) of the University of Pittsburgh Cancer Institute. Peripheral venous blood samples were obtained from HNC patients with stage III/IVA disease, receiving neoadjuvant single-agent cetuximab on a prospective phase II clinical trial (UPCI 08–013, NCT 01218048). Serum and plasma specimens were isolated from blood specimens and stored in aliquots frozen at -80 Celsius until cytokine determination. A representative sample of plasma/serum specimens from 20 patients was selected for cytokine determination. Demographics: 12 patients were randomly with age ranging from 49 to 93 years old. Samples of frozen serum specimens were thawed at room temperature for 15 minutes before starting the protocols. Human CXCL-9 (cat#DCX900, Sensitivity: 11.3 pg/mL) ELISA quantikine kits (R&D systems, Minneapolis, MN) and Human CXCL10 (cat# HCYTMAG-60K-PX29) Milliplex kit (Millipore, Minneapolis, MN) were determined according to the manufacturers protocol.

### Reagents

Recombinant human TNFα (Rhtnf-a, Invivogen/bioconnect,France), Recombinant Human Interferon-γ (11343536, Immunotools, Germany). Cetuximab (5 mg/ml; Merck serono, USA), rituximab (10 mg/ml; Roche, Switzerland), rapamycin (50 nM; Selleckchem, Germany), PD98059 (50 μM; Selleckchem), GW5074 (20 μM; Selleckchem), LY294002(25 μM; Selleckchem),SP600125 (20 μM; Selleckchem). Pamapimod (0,5uM Selleckchem), JSH-23(10uM, Selleckchem), and T-5224(20uM, Apexbt, USA). The concentration of GW5074, SP600125, LY-294,002, PD98059, Rapamycin was chosen on basis of previous results [22]. The concentration of Pamapimod (0,5uM Selleckchem), JSH-23(10uM, Selleckchem), and T-5224(20uM, Apexbt) was according to instruction of the manufacturer.

### RNA expression analysis

Total RNA was isolated using the RNeasy Plus Mini Kit (74134 Qiagen) according to the manufacturer’s instructions. Total RNA (0.5–1.0 μg) was reverse transcribed using the SuperScript III First Strand synthesis system from Invitrogen. TaqMan PCR was performed (each sample in triplicates) using TaqMan Universal PCR Master Mix and pre-designed, pre-optimized primers and probe mix for CCL2, CCL5, CXCL9, CXCL10, IL6, IL1β and GAPDH (Applied Biosystems, Foster City, USA). Threshold cycle numbers (Ct) were determined using the CFX PCR System (Bio-Rad, Veenendaal, The Netherlands), and the relative quantities of cDNA per sample were calculated using the ΔΔCt method using GAPDH as the calibrator gene.

### Western blot analysis

Polypeptides were resolved by SDS–polyacrylamide gel electrophoresis (SDS–PAGE) and transferred to a nitrocellulose membrane (Bio-Rad, Veenendaal, The Netherlands). Immune detection was achieved with primary antibodies against Phospho-c-Raf (Ser338, 9427T), Phospho-MEK1/2 (Ser217/221, 9154T), Phospho-ERK1/2 (Thr202/Tyr204, 4370T), Phospho-AKT (Ser473, 4060T), Phospho-mTOR (Ser2448, 5536T), Phospho-SAPK/JNK (Thr183/Tyr185, 4668T), Phospho-p38 MAPK (Thr180/Tyr182, 4511T), Phospho-Erk5 (Thr218/Tyr220, 3371s), IRF-1(8478s), IRF-3(11904), anti- anti-acetyl-p65 (Lys310,3045), anti-phospho-p65 (Ser536, 3033), STAT1 (#9172, CST), phospho-STAT1 (Tyr701, #9167) HRP-coupled anti-mouse (#7076s) and HRP-coupled anti-rabbit (#7074s) secondary antibodies were purchased from CST, USA. IFRD1(T2576) and β-actin (A5316) were purchased from Sigma-Aldrich. Chemoluminescence reagent (#170–5060, Bio-Rad) was used as substrate and signal was scanned using the Chemidoc and accompanying Software (Image Lab Software Version 5.2.1, Bio-Rad).

### Enzyme-linked immunosorbent assays (ELISAs) on cell supernatants

CXCL9 and CXCL10 were performed according to the manufacturer’s instruction (PeproTech, London, UK).

### Chemotaxis assays

The assay for chemotaxis was performed in 24-well plates (Costar, Cambridge, MA) carrying 6.5 mm Trans-well with 3.0 μm pore polyester membrane insert (CLS3472, Sigma). CD14-depleted PBMC were washed once and suspended at 20*10^6^ cells/ml in serum free RPMI 1640 medium. Supernatant were placed in the lower compartment, and cells were loaded onto the inserts at 2*10^6^/100μL each individual assay. Chambers were incubated for 4h in a 5% CO2-humidified incubator at 37°C. After the incubation period, numbers of CD14-depleted PBMC migrating to the lower chamber were counted by flow cytometry (BD Accuri™ C6) using counting Beads (C36950, Thermofisher) and analyzed using FlowJo v10.0.8 (Treestar, Olten, Switzerland). All conditions were tested in triplicate. Statistic evaluation was performed using the Student t test.

### Flow cytometry methods for EGFR expression

Expression of EGFR was analyzed by flow cytometry using phycoerythrin (PE)-coupled mouse-anti-human EGFR (1:20, BD Biosciences, Breda, The Netherlands). Per live gate, 50,000 cells were recorded using the BD FACS Calibur with Cellquest software (BD Bioscience) and data were analyzed using Flowjo.

### Ethics statement

This study is part of a larger study P07-112, approved by the local medical ethical committee of the Leiden University Medical Center (LUMC) and in agreement with the Dutch law.

## Results

### EGFR signaling suppresses IFNγ/TNFɑ-mediated production of immune cell attracting chemokines

**Fig 1:**
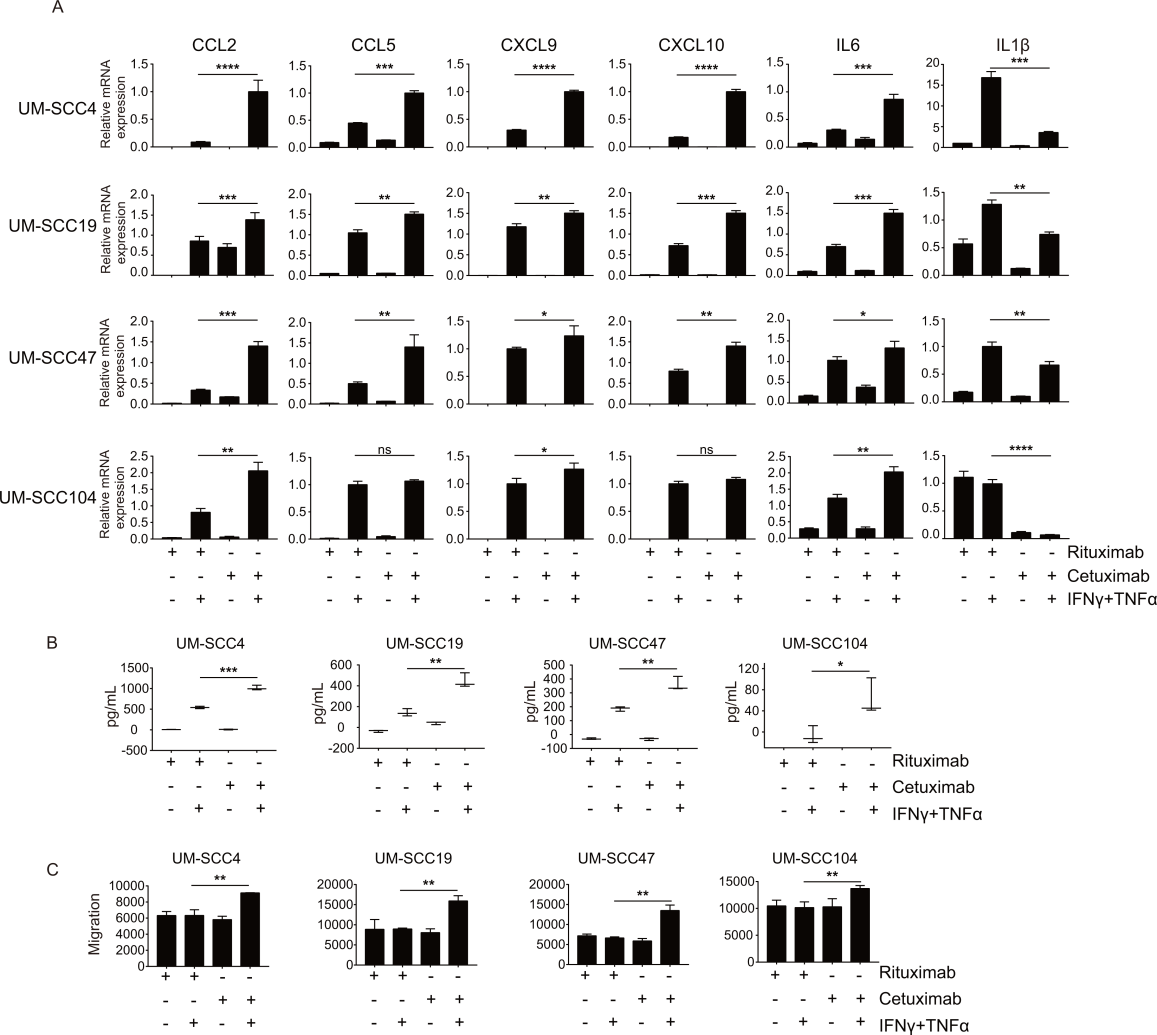
EGFR signaling suppresses IFNγ/TNFα-mediated production of immune cell attracting chemokines. Two HPV- HNC cell lines (UM-SCC4 and UM-SCC19) and two HPV+ HNC cell line (UM-SCC47 and UM-SCC104) were stimulated with 1μg/mL rituximab or 1μg/mL cetuximab as indicated. After 48h, the cells were treated with 50IU/mL IFNγ and 30ng/mL TNFα. (A)After 24h, the expression levels of the genes indicated were determined by RT-qPCR. Gene expression was normalized against GAPDH mRNA levels. Similar results were observed in two independent experiments. (B) After 48h the concentration of CXCL9 was determined by Enzyme-linked immunosorbent assay in supernatants of three different experiments. (C) The supernatant isolated after 48h were used to study CD14-depleted PBMC migration in trans well assays. P value were determined by unpaired t-tests. Ns: no significance. *P < 0.05, **P < 0.01, ***P < 0.001, ****P < 0.0001.

In order to test the effects of EGFR signaling on T cell attraction, we used the two HPV-negative UM-SCC4 and UM-SCC19 and the two HPV-positive HNSCC cell lines UM-SCC47 and UM-SCC104 for the expression of CCL2, CCL5, CXCL9, CXCL10, IL-6 and IL-1β by qPCR. All 4 cell lines display high EGFR expression (S1 Fig) and were able to signal via their IFNγ and TNFα receptors as shown by us before [9]. The cells were stimulated with or without a combination of IFNγ and TNFα as well as pre-treated with either the EGFR blocking antibody cetuximab or as control rituximab, a monoclonal antibody against CD20, for 48 hours as this will allow for functional effects of EGFR blockade on downstream targets without causing overt effects on cell density [22]. While both IFNγ and TNFα are able to increase the expression of certain chemokine genes, their combination results in even higher gene expression (S2 Fig). Treatment with the EGFR blocking antibody cetuximab alone didn’t alter the expression of cytokines. However, when the tumor cells were also stimulated with the type 1 cytokine IFNγ and TNFα an increased expression of *CCL2*, *CCL5*, *CXCL9*, *CXCL10* and *IL-6* was detected when compared to treatment with the control antibody (Fig 1A). In addition, cetuximab led to the decreased expression of *IL-1β* (Fig 1A). Several of the chemokines produced by the tumor cells, including CXCL9 and CXCL10, are important with respect to their capacity to attract T cells. Analysis of the supernatants isolated from the cultures of treated cells showed an increased amount of cytokines produced when the EGFR was blocked (Figs 1B and S3). Interestingly, the levels of these two chemokines were also found to be increased in many patients with head and neck cancers after treatment with cetuximab (S3 Fig). In addition, the tumor cell supernatant was used to study lymphocyte migration. In all 4 cases, enhanced lymphocyte infiltration was observed when PBMC where incubated with tumor cell supernatant of cetuximab treated IFNγ/TNFα stimulated cancer cells (Fig 1C). This included both CD4+ and CD8+ lymphocytes (S4 Fig). In conclusion, EGFR signaling in squamous cell carcinoma cells from the head and neck represses the production of several IFNγ/TNFα-induced T-cell attracting chemokines.

### Cetuximab blocks the activation of several downstream EGFR signaling pathways

**Fig 2:**
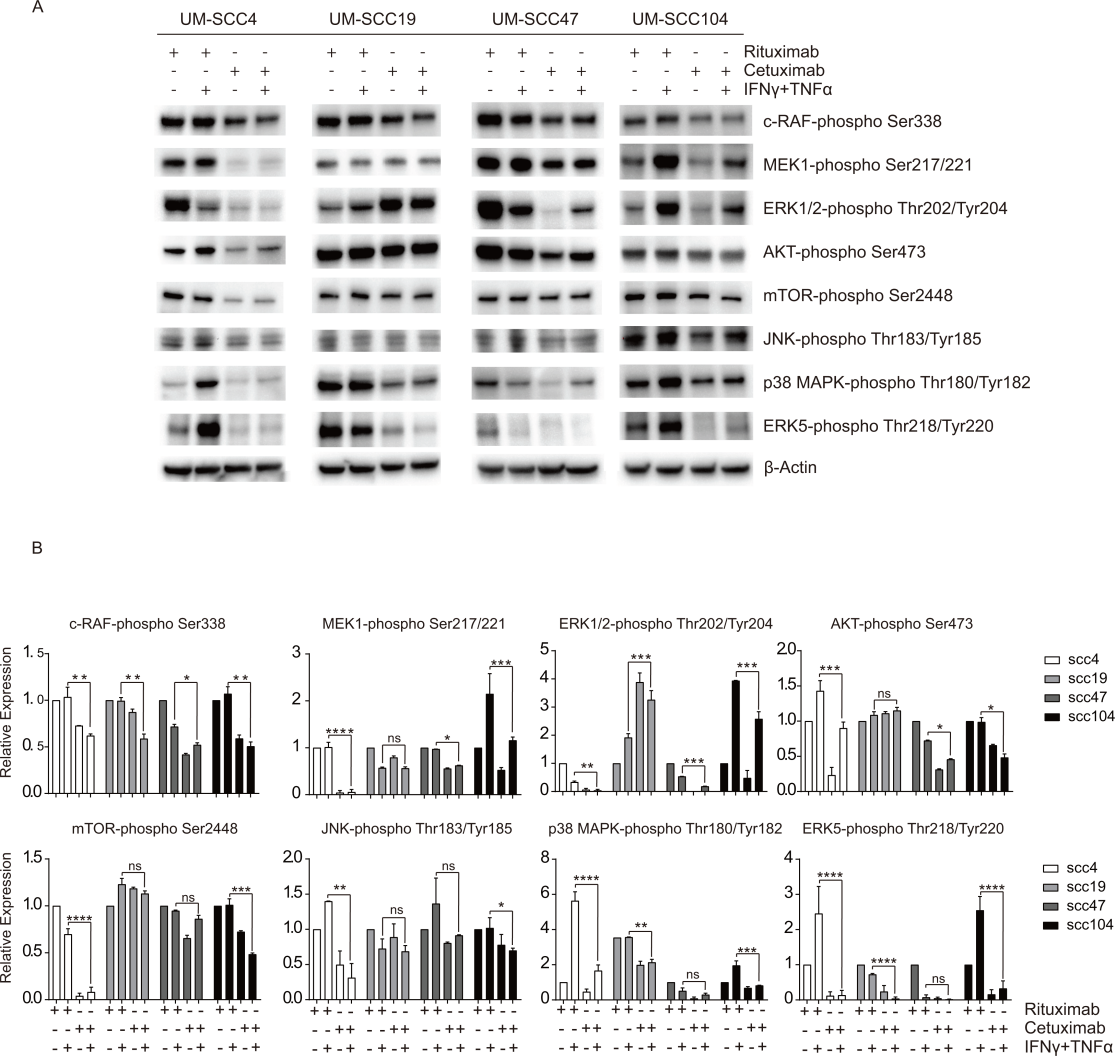
Cetuximab blocks the activation of several downstream EGFR signaling pathways. (A) Two HPV- HNC cell lines (UM-SCC4 and UM-SCC19) and two HPV+ HNC cell line (UM-SCC47 and UM-SCC104) were stimulated with 1μg/mL rituximab or 1μg/mL cetuximab as indicated. After 48h, the cells were treated with 50IU/mL IFNγ and 30ng/ml TNFα for 24h. The protein expression levels of the phosphorylated proteins were measured by Western blotting in whole cell extracts. β-actin served as loading control. (B) Relative density of proteins were quantified over β-actin. The expression levels of rituximab treatment were set as 1. Similar results were observed in two independent experiments. P value were determined by unpaired t-tests. Ns: no significance. *P < 0.05, **P < 0.01, ***P < 0.001, ****P < 0.0001.

In order to understand the mechanism underlying the repression of chemokine production by EGFR signaling, an analysis of the downstream EGFR signaling pathways was executed. The phosphorylation of the proteins in the Raf-MEK-ERK pathway, the PI3K-AKT-mTOR pathway and the MAP3K pathway was analyzed. Cetuximab treatment led to the reduction of Raf-MEK-ERK pathway and PI3K-AKT-mTOR pathway in UM-SCC4, UM-SCC47 and UM-SCC104, but not in UM-SCC19. In addition, phosphorylation of p38 and ERK5 was decreased in all cell lines upon cetuximab treatment (Fig 2).

Previously, we had found that EGFR blocking by cetuximab may also lead to the reduction of IFRD1. IFRD1 blocks enhanced cytokine production by recruiting HDAC1/3 in order to prevent acetylation of to the nucleus translocated phosphorylated p65 [22]. Therefore, also the expression and activation of multiple transcription factors was analyzed. Indeed, EGFR blocking decreased IFRD1 expression in the cell lines UM-SCC4 and UM-SCC104, but only in UM-SCC104 this resulted in increased p65 acetylation. The lack of an effect on UM-SCC4 was expected as it also failed to show p65 phosphorylation (S5 Fig). In UM-SCC47, cetuximab treatment resulted in increased STAT1 nuclear translocation and increased phosphorylation of p65 (S5 Fig) but this was not found in the other cell lines.

Interferon regulatory factors (IRF), in particular IRF1 and IRF3, have been found to regulate chemokine production [23, 24]. Therefore, IRF1 and IRF3 were knocked-down in UM-SCC4 and UM-SCC47. This showed that *CXCL10* but not *CXCL9* expression was dependent on the IRF3 in both cell lines and partly on IRF1 in UM-SCC47 (S6 Fig). No effects were seen on the expression of the other cytokines. Furthermore, cetuximab plus IFNγ/TNFα treatment of the cells had no major effects on the expression of IRF1 and IRF3 or phosphorylation of STAT1 (S5 Fig). In addition, knock-down of p65 affected the expression of *CXCL10* but had no consistent effect on the expression of the other cytokines. (S6 Fig). Moreover, we used the NFκB-inhibitor JSH-23 and AP1-inhibitor T-5224 but this did not have an impact on the gene expression of the 3 tested chemokines (S7 Fig). These data suggests that the increased levels of cytokines, in the squamous cell carcinoma cells from the head and neck, are induced by cetuximab plus IFNγ/TNFα independent of the NFκB and AP1 pathways.

Overall, these data show that blocking of the EGFR affects its downstream signaling pathways, albeit differently in individual cell lines. Furthermore, the positive effects on chemokine production observed in all cell lines could not be attributed to the down- or up-regulation of one particular signaling pathway or transcription factor.

### The MEK and JNK pathways downstream of EGFR repress chemokine expression

**Fig 3:**
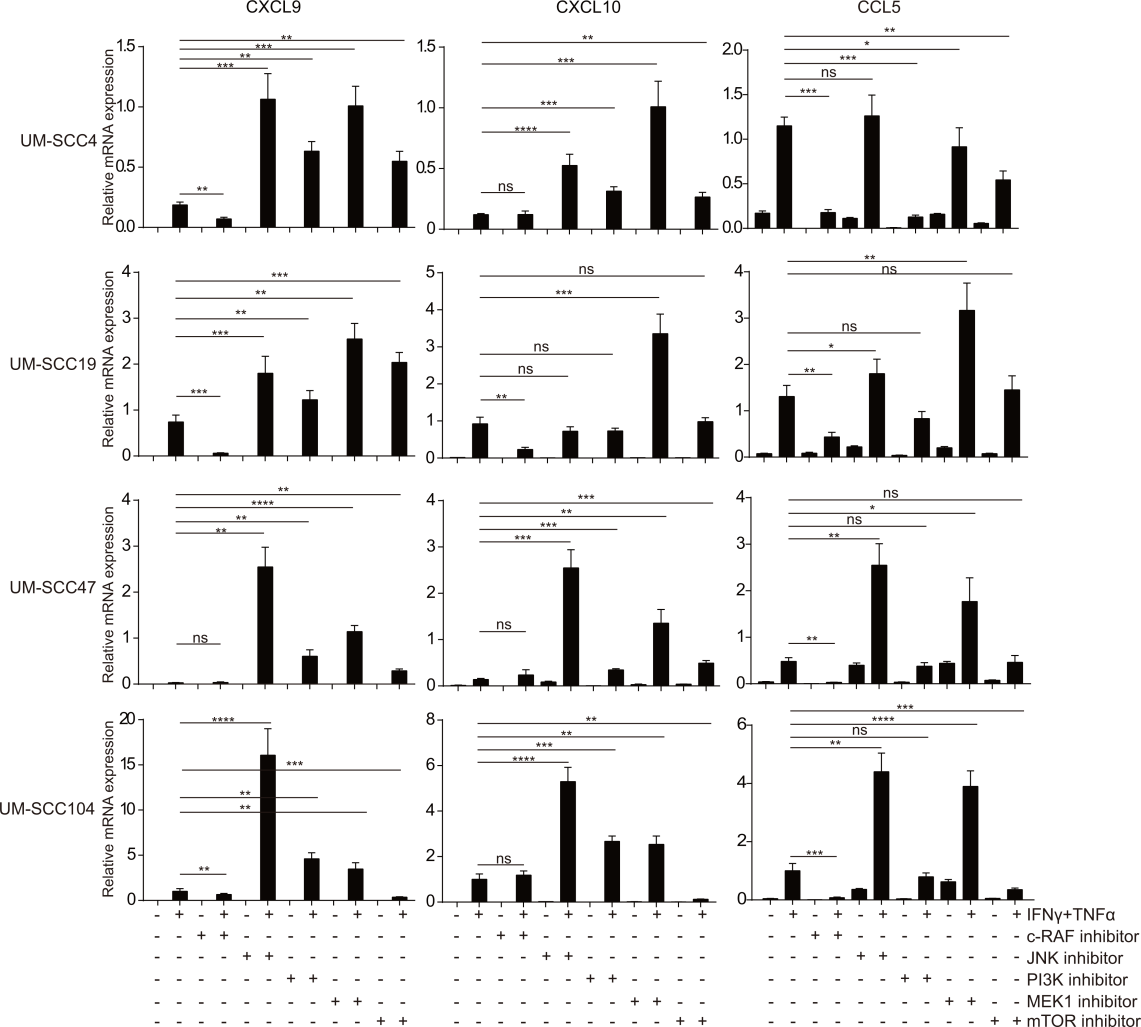
The MEK and JNK pathways downstream of EGFR repress chemokine expression. Two HPV- HNC cell lines (UM-SCC4 and UM-SCC19) and two HPV+ HNC cell line (UM-SCC47 and UM-SCC104) were stimulated with 20μM GW5074 (c-RAF inhibitor), 20μM SP600125 (JNK inhibitor), 25μM LY-294,002 (PI3K inhibitor), 50μM PD98059 (MEK1 inhibitor), 50nM Rapamycin (mTOR inhibitor) as indicated. After 48h, the cells were treated with 50IU/mL IFNγ and 30ng/ml TNFα as indicated for 24h, the expression levels of the genes indicated were determined by RT-qPCR. Gene expression was normalized against GAPDH mRNA levels. Similar results were observed in two independent experiments. P value were determined by unpaired t-tests. Ns: no significance. *P < 0.05, **P < 0.01, ***P < 0.001, ****P < 0.0001.

To mimic the effects of EGFR blocking, several chemical inhibitors of the molecules in the pathways downstream of EGFR were used followed by stimulation with IFNγ/TNFα (Fig 3). Each of the cell lines responded individually to these inhibitors but the most pronounced effects were seen when the MEK1/2 inhibitor PD98059 and the JNK inhibitor SP600125 were used. In the HPV-negative HNC cell lines UM-SCC4 and UM-SCC19, MEK1 and JNK inhibition led to the most pronounced increase in chemokine expression. In the HPV-positive HNC cell lines UM-SCC47 and UM-SCC104 blocking of JNK signaling resulted in the strongest increase of chemokine gene expression (Fig 3). The results of PI3K and mTOR inhibition were more variable between the cell lines and less pronounced when compared to MEK and JNK inhibition (Fig 3), despite the strong inhibition of downstream activation (S8 Fig). P38-signaling is known to regulate cytokine production [25], but the use of the p38 inhibitor pamapimod did not affect chemokine expression when the cells were stimulated with IFNγ/TNFα (S7 Fig). Thus, the EGFR-mediated suppression of IFNγ/TNFα induced chemokine expression in squamous cell carcinoma cells from the head and neck primarily is mediated by both the MEK and JNK signaling pathways.

### IFNγ/TNFα-induced chemokine production is c-RAF dependent

**Fig 4:**
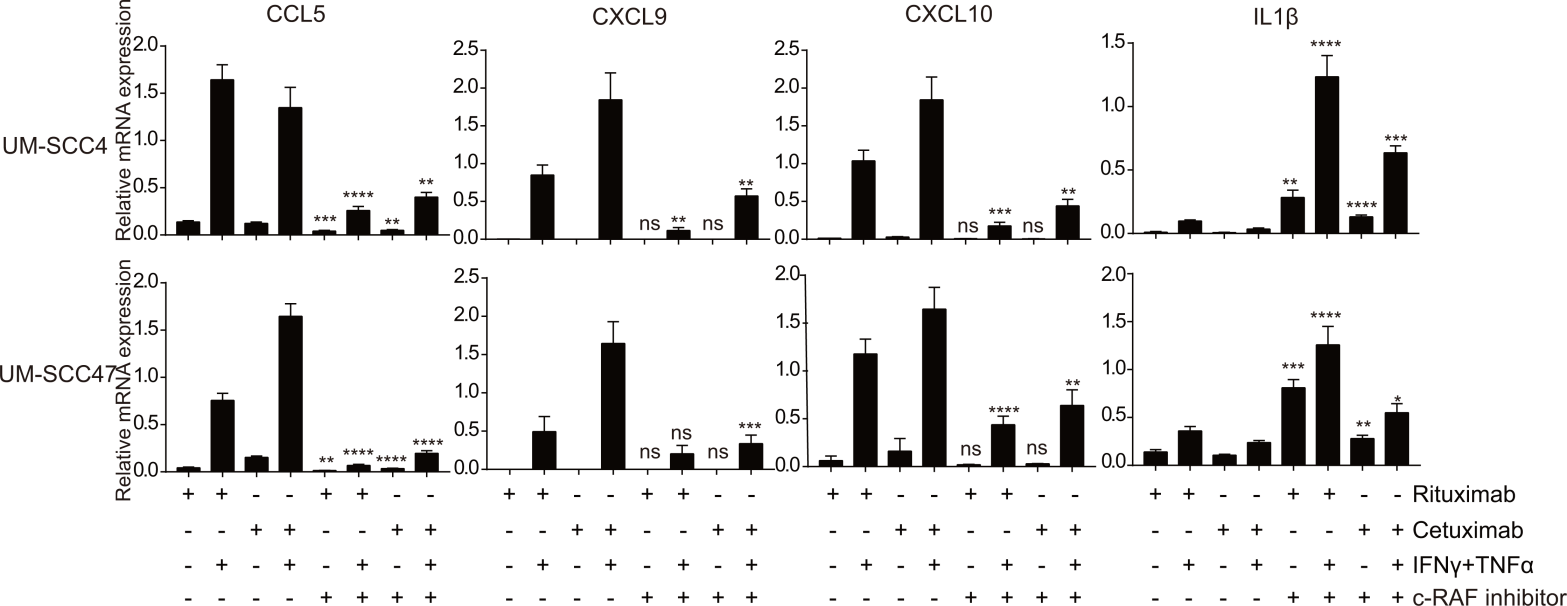
IFNγ/TNFα-induced chemokine production depends on c-RAF signalling and is not blocked by cetuximab. The HPV- HNC cell line UM-SCC4 and the HPV+ HNC cell line UM-SCC47 were stimulated with 1μg/mL rituximab or 1μg/mL cetuximab as indicated for 72h, 20 μM GW5074 (c-RAF inhibitor) as indicated for 48h, 50IU/mL IFNγ and 30ng/ml TNFα as indicated for 24h. The expression levels of the genes indicated were determined by RT-qPCR. Gene expression was normalized against GAPDH mRNA levels. Similar results were observed in two independent experiments. P value were determined by unpaired t-tests. Ns: no significance. *P < 0.05, **P < 0.01, ***P < 0.001, ****P < 0.0001.

Following EGFR signaling the RAF protein kinases become activated and translate the signal to the downstream MEK and MAP3K pathways, hence it would be logical to block this upstream target. EGFR blockade by cetuximab induced only a slightly decrease of c-RAF phosphorylation (Fig 2). However, treatment of the cell lines with the c-RAF inhibitor GW5074 resulted in a reduced expression of *CCL5*, *CXCL9* and *CXCL10* following IFNγ/TNFα stimulation in all cell lines and this negative effect could not be rescued by co-treatment of the cells with cetuximab (Fig 4). Potentially, this inhibition is related to the increased phosphorylation of ERK1/2 and MEK1 observed when the c-RAF inhibitor GW5074 was used (S8 Fig). Interestingly, blocking of c-RAF by GW5074 increased the expression levels of *IL-1β* an effect that was partly reduced by cetuximab (Fig 4). These data suggest that c-RAF signaling is a dominant factor in the IFNγ/TNFα-mediated induction of chemokine expression in the HNC cell lines. Our observations are sustained by other studies showing that activation of the IFNγR and the TNFR can result in the activation of RAF [26, 27].

### Synergistic effects of MEK and JNK inhibition with cetuximab shows that different signaling pathways act simultaneously

**Fig 5:**
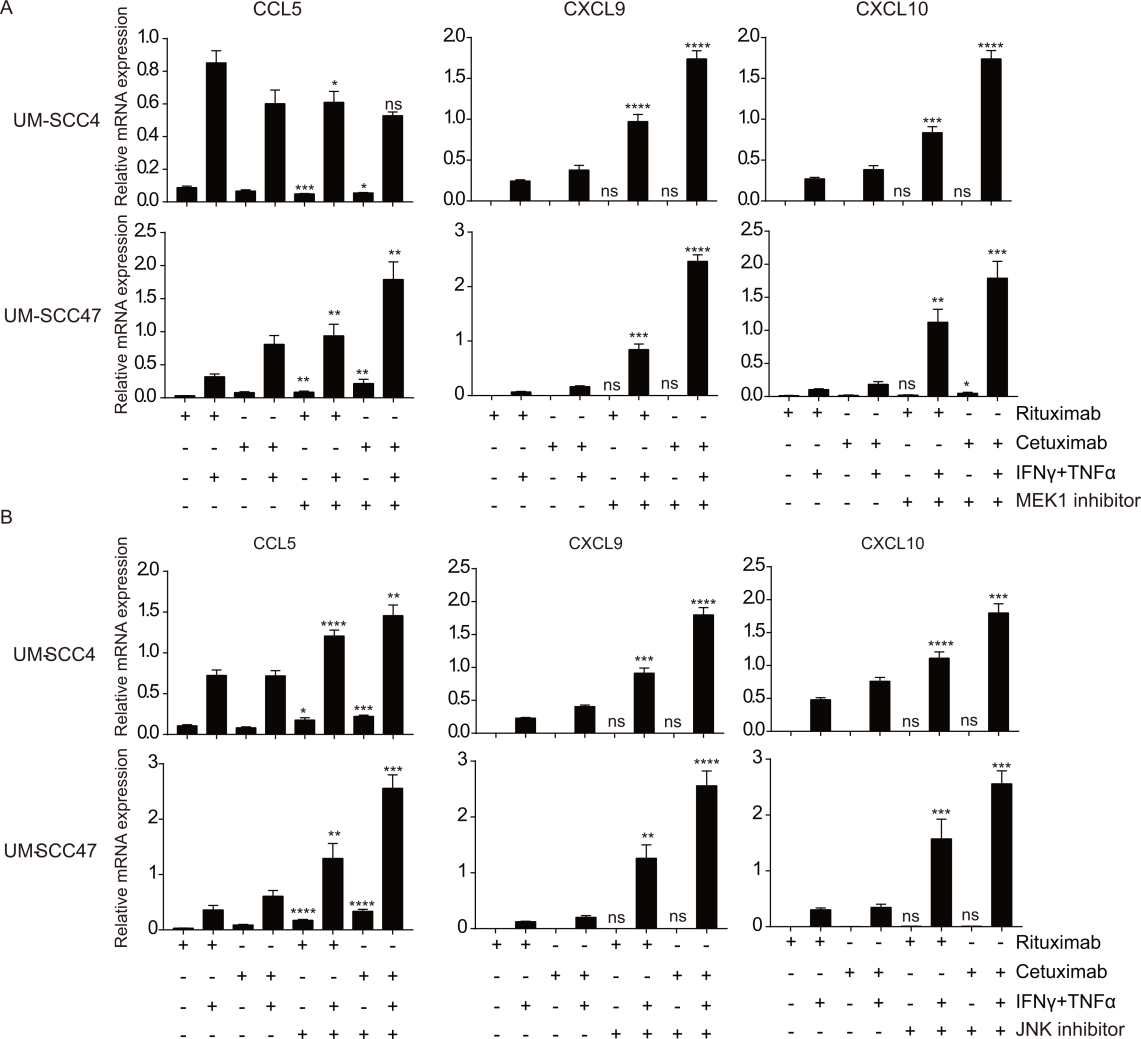
Synergistic effects of MEK and JNK inhibition with cetuximab shows that different signalling pathways act simultaneously. The HPV- HNC cell line UM-SCC4 and the HPV+ HNC cell line UM-SCC47 were stimulated with 1μg/mL rituximab or 1μg/mL cetuximab as indicated for 72h, a specific inhibitor as indicated for 48h, 50IU/mL IFNγ and 30ng/ml TNFα as indicated for 24h. The expression levels of the genes indicated were determined by RT-qPCR. Gene expression was normalized against GAPDH mRNA levels. (A)50μM of PD98059 (MEK1 inhibitor) was used. (B)20μM of SP600125 (JNK inhibitor) was used. Similar results were observed in two independent experiments. P value were determined by unpaired t-tests. Ns: no significance. *P < 0.05, **P < 0.01, ***P < 0.001, ****P < 0.0001.

**Fig 6:**
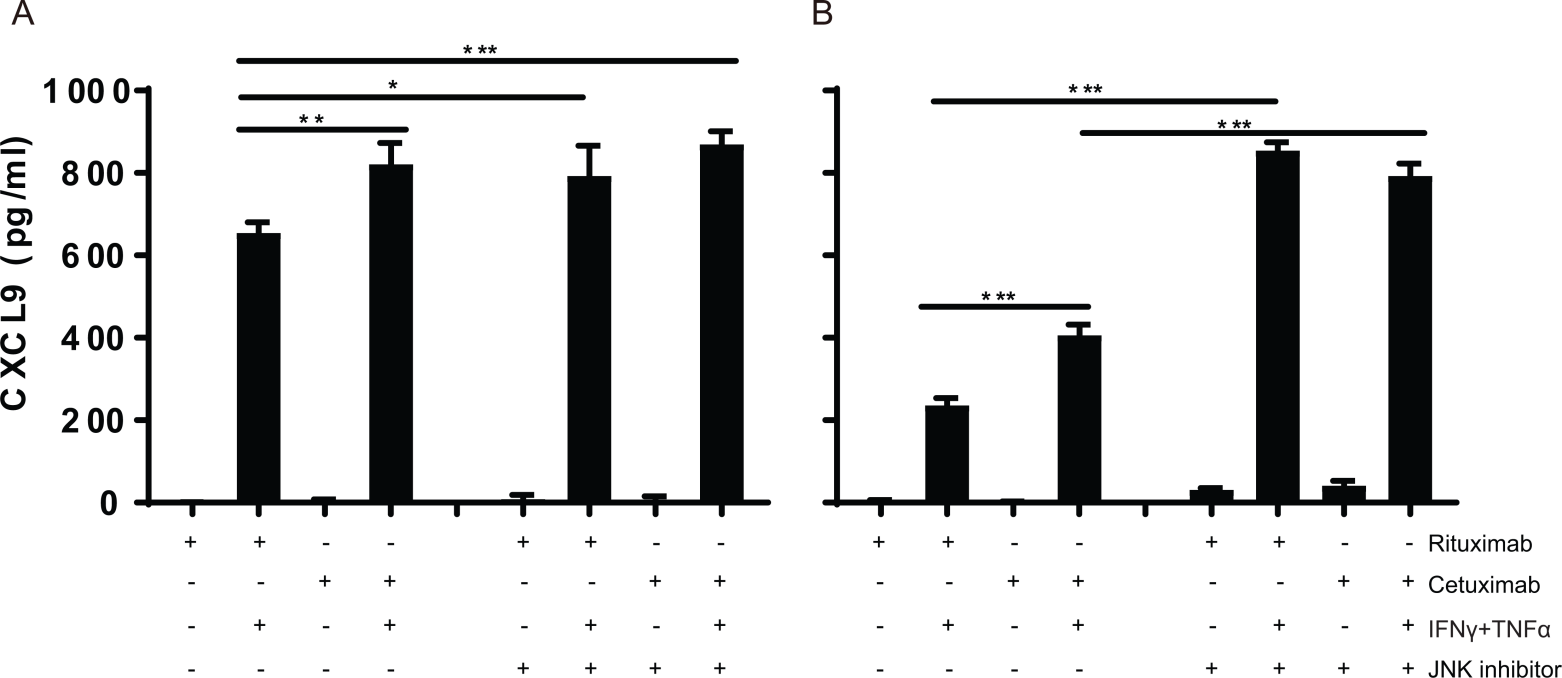
JNK inhibition increases the production of CXCL9 in IFNγ/TNFα-stimulated tumor cells. The cell lines A) UM-SCC4 and B) UM-SCC47 were stimulated with 1μg/mL rituximab or 1μg/mL cetuximab as indicated for 72h, and the JNK specific inhibitor SP600125 as indicated for 48h, 50IU/mL IFNγ and 30ng/ml TNFα as indicated for 24h. After 48h the concentration of CXCL9 was determined by Enzyme-linked immunosorbent assay in the supernatants P value were determined by unpaired t-tests. Ns: no significance. *P < 0.05, **P < 0.01, ***P < 0.001, ****P < 0.0001.

**Fig 7:**
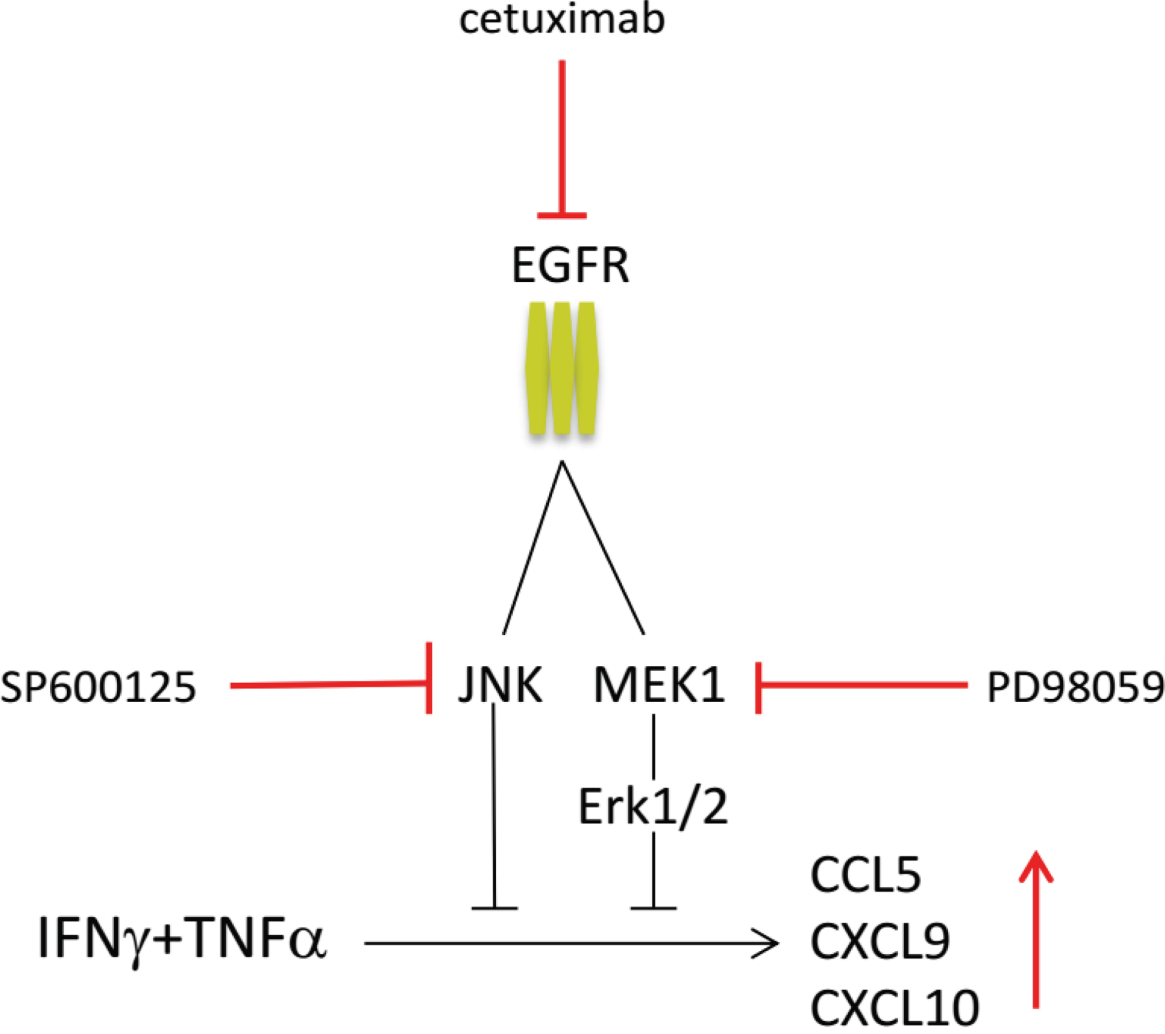
Schematic representation of EGFR mediated repression of chemokine production. EGFR signalling suppresses IFNγ/TNFα related production of T cell attracting cytokines, including CCL5, CXCL9 and CXCL10 mainly through activation of JNK and MEK1 signaling. Inhibition of EGFR signaling with cetuximab, JNK inhibitor SP600125 and/or MEK1 inhibitor PD98059, restores the IFNγ/TNFα stimulated production of T cell attracting cytokines.

In order to test if blocking of MEK1, JNK or mTOR downstream of EGFR would mimic the results obtained when treating the cells with cetuximab, the tumor cells were incubated with cetuximab and/or one of the chemical inhibitors PD98059 (MEK), SP600125 (JNK) and Rapamycin (mTOR) and then stimulated with IFNγ/TNFα. Blocking of MEK1 or JNK resulted in an enhanced expression of all three chemokines to a level that was similar or higher to those seen when cetuximab alone was used (Fig 5A and 5B). When the cells were treated with a combination of cetuximab and MEK or JNK, the expression levels of *CCL5*, *CXCL9* and *CXCL10* increased, confirming that not one but multiple pathways downstream of the EGFR act in concert to block IFNγ/TNFα -induced chemokine expression by these cancer cell lines. At the protein level, JNK inhibition of IFNγ/TNFα stimulated UM-SCC4 and UM-SCC47 cells resulted in an increased secretion of CXCL9 to a level that was not increased by additional EGFR blocking using cetuximab (Fig 6). Notably, blocking of mTOR again had variable effects and failed to recapitulate the effect of cetuximab on *CXCL9*. In addition, the levels of the chemokines did not always increase when the cells were treated with cetuximab and the mTOR inhibitor when compared to treatment with cetuximab only (S7 Fig). In conclusion, overexpression of EGFR results in the activation of the JNK and MEK signaling pathways that act simultaneously to downregulate the IFNγ/TNFα production of chemokines required to attract T cells (Fig 7).

## Discussion

In this study, we analyzed the immune regulatory properties of EGFR signaling in head and neck cancer cells and showed that it suppresses type 1 cytokine-induced expression of *CCL2*, *CCL5*, *CXCL9*, *CXCL10* and *IL-6* while promoting the expression of *IL-1β*. Blocking of the EGFR with cetuximab when tumor cells were stimulated with the type 1 cytokine IFNγ and TNFα resulted in the amplification of the production of the T-cell attracting chemokines and resulted in an increased migration of CD4+ and CD8+ lymphocytes in chemotaxis assays in vitro. In vivo, cetuximab treatment enhanced the serum levels of CXCL9 and CXCL10 in patients with head and neck cancer. Our data are in line with previous studies showing that EGFR mutated tumors display an uninflamed phenotype [17] and that cetuximab treatment may lead to increased T-cell infiltration in head and neck cancers [28, 29]. Whilst others observed that EGFR inhibition can lead to the direct increased secretion of proinflammatory cytokines, including IL2, IL4, IL6, IL8, GMCSF [30], our study showed an increased expression of CCL2, CCL5, CXCL9, CXCL10 after EGFR blockade only when the tumor cells were stimulated with IFNγ and TNFα. Mechanistic studies revealed that the EGFR mediated the repression of chemokine production through the activation of multiple downstream signaling pathways, predominantly via JNK and MEK1, that act simultaneously. In melanoma, the B-raf, β-catenin and PTEN-PI3K/mTOR oncogenic pathways have been identified as direct repressors of tumor-infiltration by T cells [20, 21, 31]. We show that the EGFR-signaling pathway complements this list albeit that it acts a bit different in that it suppresses the type 1 cytokine mediated amplification of tumor infiltration by T cells, via blockade of the production of T-cell attracting chemokine by tumor cells when stimulated with type 1 cytokines.

Interestingly, blocking of the downstream molecule c-RAF by GW5074 blunted the IFNγ/TNFα induced expression of the T-cell attracting chemokines while it enhanced the expression of *IL-1β* confirming earlier reports that RAF can be activated by both IFNγ [27, 32] and TNFα [33], and revealing an important role for c-RAF in relaying the signals induced by IFNγ and TNFα that lead to cytokine production. The enhanced expression of *IL-1β* after GW5074 treatment was related to an increased phosphorylation of MEK1 and ERK1/2, indirectly confirming that activation of EGFR-downstream MEK and ERK affect IFNγ/TNFα induced chemokine secretion in head and neck cancer. Blocking of the EGFR by cetuximab enhanced the expression of the T-cell attracting chemokines but lowered the expression of *IL-1β* Potentially, this contributes to the intratumoral T cell responses as IL-1β may indirectly suppress the proliferation and function of these T cells via tumor associated fibroblasts that start to express COX-2 and PD-L1 [34]. In addition, IL-1β induces the accumulation of immune suppressive myeloid derived suppressor cells [35]. Thus, cetuximab treatment may not only amplify the attraction of T cells but also allow for the accumulation of more effective T cells in the tumor.

T cell infiltration of head and neck cancers is a positive prognostic factor in head and neck cancer [2–4, 36]. The EGFR-mediated suppression of IFNγ/TNFα mediated amplification of the T-cell infiltrate in these cancers bears strong similarities with observations in skin inflammation disorders. Also, here IFNγ and TNFα promote *de novo* synthesis of numerous chemokines responsible for the attraction of immune cells but this reaction is suppressed by active EGFR signaling. Blockade of the EGFR pathway increases chemokine production and leads to enhanced immune cell infiltration of the skin [37] Interestingly, in these skin disorders the activation of EGFR was the result of increased levels of soluble EGFR ligands produced by keratinocytes in response to IFNγ and TNFα present in the milieu [37], suggesting that EGFR-mediated suppression of chemokine production can occur because of constitutive EGFR-signaling but also as result of an adaptive negative feed-back loop. Other reported immune escape mechanisms that can play a role because of constitutive or adaptive EGFR-signaling are the expression of PD-L1 and the inhibition of antigen processing via dephosphorylation of STAT1 in cancer cells [16, 38, 39]. Notably, in one (UM-SCC47) of the 4 HNSCC cell lines studied cetuximab treatment increased the level of phosphorylated STAT1 in the nucleus.

In order to study the mechanism underlying EGFR-mediated suppression of chemokine expression we analyzed its downstream pathways. Previously, we found that EGFR was overexpressed in HPV-infected keratinocytes and resulted in the upregulation of interferon-related developmental regulator 1 (IFRD1) which suppressed IFNγ/TNFα induced chemokine expression by preventing the acetylation of RelA [40]. The involvement of IFRD1 was also studied in the HPV-negative and HPV-positive cell lines used in this study. Potentially it plays an additional role in one (UM-SCC104) of the 4 cell lines but not in the other cell lines. Furthermore, we found quite a variability in the expression and phosphorylation of the proteins downstream of the EGFR. However, in combination with the data obtained when specific pathway inhibitors were used it became clear that in all cell lines the suppression of chemokine production was mediated via the EGFR downstream molecules MEK1 and JNK. One possible acting mechanism is the destabilization of the newly synthesized mRNAs via the activation of MEK/ERK [41]. Interestingly, sustained ERK activation is also a part of the normal regulatory pathway of IFNγ signaling mediated by the suppressor of cytokine signaling 1 [32]. Another reported MEK related mechanism influencing chemokine expression is the enhanced nuclear translocation of IRF-1 [23, 42], but this was not observed by us. A third non-exclusively occurring MEK/ERK-related mechanism which may play a role is the ERK negative feedback phosphorylation that inactivates RAF signaling [43], which we have shown is important for relaying the signals of the IFNγR and TNFR in our system. Inhibition of EGFR signaling has also been reported to stimulate the expression and activation of NOX4 resulting in hydrogen peroxide-induced oxidative stress, consequently leading to activation of NFκB and AP-1 with as result the induction of pro-inflammatory cytokines. This mechanism is not likely to play a role in the suppression of IFNγ/TNFα mediated chemokine production as inhibition of AP1 or NFκB did not influence the expression levels of the tested chemokines. The downstream molecules P38 and JNK play a crucial role in biosynthesis of cytokines [44]. Our results showed no role for P38 while JNK did influence IFNγ/TNFα mediated chemokine production. Especially in the HPV+ HNC cell lines the impact of JNK inhibition was stronger than that of MEK/ERK inhibition. JNK knock-out macrophages also display higher levels of the chemokines *CCL5*, *CXCL9* and *CXCL10* after stimulation with IFNγ and potentially this is related to modulation of the mRNA expression levels [45], suggesting that similar to MEK/ERK also JNK may regulate chemokine expression in the head and neck cancer cells by controlling mRNA stability.

In conclusion, we provided evidence that overexpression of EGFR in head and neck cancer cells results in the activation of multiple downstream signaling pathways that act simultaneously to repress the type 1 cytokine mediated amplification of tumor T cell infiltration by suppressing the production of T-cell attracting chemokines. Our experiments highlight a dominant role for the downstream MEK and JNK pathways in this process. Interfering with this process may increase the efficacy of current T-cell based immunotherapies, not only in head and neck cancer but also in several others types of cancer where overexpression of EGFR is found. Direct inhibition of MEK and JNK via targeted therapy is not likely to help the antitumor response as these pathways are also important for the proliferation, polarization and cytotoxic capacity of T cells [44], leaving direct blocking of the EGFR as the best possible current option.

## Supporting information

S1 Fig EGFR surface protein expression. Histograms of EGFR expression by two HPV- HNC cell lines UM-SCC4 and UM-SCC9 and two HPV+ HNC cell lines UM-SCC47 and UM-SCC104 as determined by flow cytometry. TC-1 is the mouse cell line that served as negative control and which can’t be stained by the antibody, HPV16+ keratinocytes served as the positive control.
(EPS)

S2 Fig Chemokine expression after cetuximab treatment. HPV- HNC cell line UM-SCC4 and HPV+ HNC cell line UM-SCC47 were stimulated with 1μg/mL rituximab or1μg/mL cetuximab as indicated. After 48h, the cells were treated with 50IU/mL IFNγ and/or 30ng/ml TNFα as indicated for 24h, The expression levels of genes as indicated were determined by RT-qPCR. Gene expression was normalized against GAPDH mRNA levels. Similar results were observed in two independent experiments. P values were determined by unpaired t-tests. Ns: not significant. *P < 0.05, **P < 0.01, ***P < 0.001, ****P < 0.0001.
(EPS)

S3 Fig Chemokine production. (A)Two HPV- HNC cell lines (UM-SCC4 and UM-SCC19) and two HPV+ HNC cell lines (UM-SCC47 and UM-SCC104) were stimulated with 1μg/mL rituximab or 1μg/mL cetuximab as indicated. After 24h, the cells were treated with 50IU/mL IFNγ and 30ng/ml TNFα as indicated for 48h. The OD value in supernatants of CXCL9 and CXCL10 was determined by Enzyme-linked immunosorbent assay. P values were determined by unpaired t-tests. Ns: not significant. *P < 0.05, **P < 0.01, ***P < 0.001, ****P < 0.0001. (B) Peripheral venous blood samples were obtained from HNC patients with stage III/IVA disease, receiving neoadjuvant single-agent cetuximab in a prospective phase II clinical trial. A representative pre- and post-treatment sample from 12 randomly selected patients (all Caucasian, age 49–93 years old) were used for cytokine determination.
(EPS)

S4 Fig Enhanced migration of T cells after cetuximab treatment. UM-SCC4 was stimulated with 1μg/mL rituximab or 1μg/mL cetuximab as indicated. After 24h, the cells were treated with 50IU/mL IFNγ and 30ng/ml TNFα as indicated for 48h. CD14-depleted PBMCs migration towards supernatants was determined by trans well assay. The number of CD4+ and CD8+ T cells within migrated CD14-depleted PBMC was determined by flow cytometry. P values were determined by unpaired t-tests. Ns: not significant. *P < 0.05, **P < 0.01, ***P < 0.001, ****P < 0.0001.
(EPS)

S5 Fig Biochemical analyses of signalling pathways. (A) Two HPV- HNC cell lines (UM-SCC4 and UM-SCC19) and two HPV+ HNC cell lines (UM-SCC47 and UM-SCC104) were stimulated with 1μg/mL rituximab or 1μg/mL cetuximab as indicated. After 48h, the cells were treated with 50IU/mL IFNγ and 30ng/mL TNFα as indicated for 24h. The protein expression levels of IRF1, IRF3, IFRD1, p65-acetylation, p65-phosphorylation, phosphor-STAT1 Tyr701 and Phospho-STAT1 Ser727 as detected by Western blotting (WB) in whole cell extracts. β-actin served as loading control. (B)Two HPV- HNC cell lines (UM-SCC4 and UM-SCC19) and two HPV+ HNC cell lines (UM-SCC47 and UM-SCC104) were stimulated with 1ug/mL rituximab or 1ug/mL cetuximab as indicated. After 48h, the cells were treated with 50IU/mL IFNγ, 30ng/ml TNFα as indicated for 24h. The protein expression levels of IRF1, IRF3, p65, STAT1 as detected by Western blotting (WB) in nuclear extracts is shown. Histone3 served as loading control.
(EPS)

S6 Fig Chemokine expression after blockade of signalling pathway proteins IRF1, IRF3 or p65. (A,B) Expression of *CXCL9* and *CXCL10* in HPV- HNC cell line UM-SCC4 and HPV+ HNC cell line UM-SCC47 transfected with control siRNA (siControl) or siRNA targeting IRF1 or IRF3 stimulated with or without 1μg/mL rituximab or 1μg/mL cetuximab as indicated. After 24h, the cells were treated with 50IU/mL IFNγ and 30ng/ml TNFα as indicated for 24h. Gene expression was normalized against GAPDH mRNA levels and standardized against siControl. Similar results were observed in two independent experiments. P value were determined by unpaired t-tests of siControl group compared with siIRF1 and siIRF3 group, respectively. Ns: not significant. *P < 0.05, **P < 0.01, ***P < 0.001, ****P < 0.0001. (C) Expression of *CCL5*, *CXCL9* and *CXCL10* in HPV- HNC cell line UM-SCC4 and HPV+ HNC cell line UM-SCC47 transfected with control siRNA (siControl) or siRNA targeting P65 stimulated with or without 1ug/mL rituximab or 1ug/mL cetuximab as indicated. After 24h, the cells were treated with 50IU/mL IFNγ and 30ng/ml TNFα as indicated for 24h. Gene expression was normalized against GAPDH mRNA levels and standardized against siControl. Similar results were observed in two independent experiments. P values were determined by unpaired t-tests of siControl group compared with siIRF1 and siIRF3 group respectivley. Ns: not significant. *P < 0.05, **P < 0.01, ***P < 0.001, ****P < 0.0001.
(EPS)

S7 Fig Chemokine expression after blockade of signalling pathway proteins AP1, NFκB, p38 or mTOR. HPV- HNC cell line UM-SCC4 and HPV+ HNC cell line UM-SCC47 were stimulated with1μg/mL rituximab or 1μg/mL cetuximab as indicated for 72h, (A)10μM JSH-23 (NFκB inhibitor) and 20μM T-5224 (AP-1 inhibitor), or (B) 0,5 μM pamapimod (P38 inhibitor), or (C) 50nM Rapamycin(mTOR inhibitor) as indicated for 48h, 50IU/mL IFNγ and 30ng/ml TNFα as indicated for 24h. The expression levels of CCL5, CXCL9 and CXCL10 were determined by RT-qPCR. Gene expression was normalized against GAPDH mRNA levels. Similar results, were observed in two independent experiments.
(EPS)

S8 Fig Signalling pathway inhibitor controls. UM-SCC4 was stimulated with (A) 20 μM SP600125 (JNK inhibitor), (B) 50μM PD98059 (MEK1 inhibitor), (C) 50nM Rapamycin (mTOR inhibitor), (D) 25 μM LY-294,002 (PI3K inhibitor), (E)20μM GW5074 (c-RAF inhibitor) for 2h, then the cells were treated with 50IU/mL IFNγ and 30ng/ml TNFα for different time points as indicated. The expression levels of proteins as indicated were detected by Western blotting (WB) in whole cell extracts. β-actin served as loading control. Similar results were observed in two independent experiments.
(EPS)

## References

[1] Sankaranarayanan R, Masuyer E, Swaminathan R, Ferlay J, Whelan S. Head and neck cancer: a global perspective on epidemiology and prognosis. Anticancer research. 1998;18(6B):4779–86. 9891557

[2] Nguyen N, Bellile E, Thomas D, McHugh J, Rozek L, Virani S, et al Tumor infiltrating lymphocytes and survival in patients with head and neck squamous cell carcinoma. Head & neck. 2016;38(7):1074–84. 26879675 10.1002/hed.24406 PMC4900934

[3] Kim HR, Ha S-J, Hong MH, Heo SJ, Koh YW, Choi EC, et al PD-L1 expression on immune cells, but not on tumor cells, is a favorable prognostic factor for head and neck cancer patients. Scientific reports. 2016;6:36956 10.1038/srep36956 27841362 PMC5107906

[4] Balermpas P, Michel Y, Wagenblast J, Seitz O, Weiss C, Rödel F, et al Tumour-infiltrating lymphocytes predict response to definitive chemoradiotherapy in head and neck cancer. British journal of cancer. 2014;110(2):501–9. 10.1038/bjc.2013.640 24129245 PMC3899751

[5] Ang KK, Harris J, Wheeler R, Weber R, Rosenthal DI, Nguyen-Tân PF, et al Human papillomavirus and survival of patients with oropharyngeal cancer. New England Journal of Medicine. 2010;363(1):24–35. 10.1056/NEJMoa0912217 20530316 PMC2943767

[6] Wansom D, Light E, Worden F, Prince M, Urba S, Chepeha DB, et al Correlation of cellular immunity with human papillomavirus 16 status and outcome in patients with advanced oropharyngeal cancer. Archives of Otolaryngology–Head & Neck Surgery. 2010;136(12):1267–73. 21173378 10.1001/archoto.2010.211 PMC3342998

[7] Badoual C, Hans S, Rodriguez J, Peyrard S, Klein C, Agueznay NEH, et al Prognostic value of tumor-infiltrating CD4+ T-cell subpopulations in head and neck cancers. Clinical cancer research. 2006;12(2):465–72. 10.1158/1078-0432.CCR-05-1886 16428488

[8] Badoual C, Hans S, Merillon N, Van Ryswick C, Ravel P, Benhamouda N, et al PD-1–expressing tumor-infiltrating T cells are a favorable prognostic biomarker in HPV-associated head and neck cancer. Cancer research. 2013;73(1):128–38. 10.1158/0008-5472.CAN-12-2606 23135914

[9] Welters MJ, Ma W, Santegoets SJ, Goedemans R, Ehsan I, Jordanova ES, et al Intratumoral HPV16-specific T-cells constitute a type 1 oriented tumor microenvironment to improve survival in HPV16-driven oropharyngeal cancer. Clinical Cancer Research. 2017:clincanres. 2140.017. 10.1158/1078-0432.CCR-17-2140 29018052

[10] Ferris RL, Blumenschein G Jr, Fayette J, Guigay J, Colevas AD, Licitra L, et al Nivolumab for recurrent squamous-cell carcinoma of the head and neck. The New England journal of medicine. 2016;2016(375):1856–67. 10.1056/NEJMoa1602252 PMC5564292 27718784

[11] Maiti GP, Mondal P, Mukherjee N, Ghosh A, Ghosh S, Dey S, et al Overexpression of EGFR in head and neck squamous cell carcinoma is associated with inactivation of SH3GL2 and CDC25A genes. PloS one. 2013;8(5):e63440 10.1371/journal.pone.0063440 23675485 PMC3651136

[12] Yunhong T, Jie L, Yunming T, Guoqian Z, Xing Z, Ronghui Z, et al Efficacy and safety of anti‐EGFR agents administered concurrently with standard therapies for patients with head and neck squamous cell carcinoma: A systematic review and meta‐analysis of randomized controlled trials. International journal of cancer. 2017. 10.1002/ijc.31157 29143328

[13] Tejani MA, Cohen RB, Mehra R. The contribution of cetuximab in the treatment of recurrent and/or metastatic head and neck cancer. Biologics: targets & therapy. 2010;4:173. 20714355 10.2147/btt.s3050 PMC2921255

[14] Pollack BP, Sapkota B, Cartee TV. Epidermal growth factor receptor inhibition augments the expression of MHC class I and II genes. Clinical Cancer Research. 2011;17(13):4400–13. 10.1158/1078-0432.CCR-10-3283 21586626

[15] Akbay EA, Koyama S, Carretero J, Altabef A, Tchaicha JH, Christensen CL, et al Activation of the PD-1 pathway contributes to immune escape in EGFR-driven lung tumors. Cancer discovery. 2013;3(12):1355–63. 10.1158/2159-8290.CD-13-0310 24078774 PMC3864135

[16] Concha-Benavente F, Srivastava RM, Trivedi S, Lei Y, Chandran U, Seethala RR, et al Identification of the cell-intrinsic and-extrinsic pathways downstream of EGFR and IFNγ that induce PD-L1 expression in head and neck cancer. Cancer research. 2016;76(5):1031–43. 10.1158/0008-5472.CAN-15-2001 26676749 PMC4775348

[17] Dong Z-Y, Zhang J-T, Liu S-Y, Su J, Zhang C, Xie Z, et al EGFR mutation correlates with uninflamed phenotype and weak immunogenicity, causing impaired response to PD-1 blockade in non-small cell lung cancer. OncoImmunology. 2017;6(11):e1356145 10.1080/2162402X.2017.1356145 29147605 PMC5674946

[18] Chong CR, Jänne PA. The quest to overcome resistance to EGFR-targeted therapies in cancer. Nature medicine. 2013;19(11):1389–400. 10.1038/nm.3388 24202392 PMC4049336

[19] Peng W, Chen JQ, Liu C, Malu S, Creasy C, Tetzlaff MT, et al Loss of PTEN promotes resistance to T cell–mediated immunotherapy. Cancer discovery. 2016;6(2):202–16. 10.1158/2159-8290.CD-15-0283 26645196 PMC4744499

[20] Liu C, Peng W, Xu C, Lou Y, Zhang M, Wargo JA, et al BRAF inhibition increases tumor infiltration by T cells and enhances the antitumor activity of adoptive immunotherapy in mice. Clinical cancer research. 2013;19(2):393–403. 10.1158/1078-0432.CCR-12-1626 23204132 PMC4120472

[21] Spranger S, Bao R, Gajewski TF. Melanoma-intrinsic [bgr]-catenin signalling prevents anti-tumour immunity. Nature. 2015;523(7559):231–5. 10.1038/nature14404 25970248

[22] Tummers B, Goedemans R, Pelascini LP, Jordanova ES, Van Esch EM, Meyers C, et al The interferon-related developmental regulator 1 is used by human papillomavirus to suppress NFκB activation. Nature communications. 2015;6:6537 10.1038/ncomms7537 26055519 PMC4382698

[23] Zaheer RS, Koetzler R, Holden NS, Wiehler S, Proud D. Selective transcriptional down-regulation of human rhinovirus-induced production of CXCL10 from airway epithelial cells via the MEK1 pathway. The Journal of Immunology. 2009;182(8):4854–64. 10.4049/jimmunol.0802401 19342664

[24] Carrigan SO, Junkins R, Yang YJ, MacNeil A, Richardson C, Johnston B, et al IFN regulatory factor 3 contributes to the host response during Pseudomonas aeruginosa lung infection in mice. The Journal of Immunology. 2010;185(6):3602–9. 10.4049/jimmunol.0903429 20720199

[25] Wagner EF, Nebreda ÁR. Signal integration by JNK and p38 MAPK pathways in cancer development. Nature Reviews Cancer. 2009;9(8):537–49. 10.1038/nrc2694 19629069

[26] Belka C, Wiegmann K, Adam D, Holland R, Neuloh M, Herrmann F, et al Tumor necrosis factor (TNF)-alpha activates c-raf-1 kinase via the p55 TNF receptor engaging neutral sphingomyelinase. The EMBO journal. 1995;14(6):1156 7720706 10.1002/j.1460-2075.1995.tb07099.x PMC398193

[27] Zibara K, Zeidan A, Bjeije H, Kassem N, Badran B, El-Zein N. ROS mediates interferon gamma induced phosphorylation of Src, through the Raf/ERK pathway, in MCF-7 human breast cancer cell line. Journal of cell communication and signaling. 2017;11(1):57–67. 10.1007/s12079-016-0362-6 27838900 PMC5362574

[28] Jie H-B, Schuler PJ, Lee SC, Srivastava RM, Argiris A, Ferrone S, et al CTLA-4+ regulatory T cells increased in cetuximab-treated head and neck cancer patients suppress NK cell cytotoxicity and correlate with poor prognosis. Cancer research. 2015;75(11):2200–10. 10.1158/0008-5472.CAN-14-2788 25832655 PMC4452385

[29] Trivedi S, Srivastava RM, Concha-Benavente F, Ferrone S, Garcia-Bates TM, Li J, et al Anti-EGFR targeted monoclonal antibody isotype influences antitumor cellular immunity in head and neck cancer patients. Clinical Cancer Research. 2016;22(21):5229–37. 10.1158/1078-0432.CCR-15-2971 27217441 PMC5093040

[30] Fletcher EV, Love-Homan L, Sobhakumari A, Feddersen CR, Koch AT, Goel A, et al EGFR inhibition induces proinflammatory cytokines via NOX4 in HNSCC. Molecular Cancer Research. 2013;11(12):1574–84. 10.1158/1541-7786.MCR-13-0187 24048704 PMC3869882

[31] Frederick DT, Piris A, Cogdill AP, Cooper ZA, Lezcano C, Ferrone CR, et al BRAF inhibition is associated with enhanced melanoma antigen expression and a more favorable tumor microenvironment in patients with metastatic melanoma. Clinical cancer research. 2013;19(5):1225–31. 10.1158/1078-0432.CCR-12-1630 23307859 PMC3752683

[32] Madonna S, Scarponi C, De Pità O, Albanesi C. Suppressor of cytokine signaling 1 inhibits IFN-γ inflammatory signaling in human keratinocytes by sustaining ERK1/2 activation. The FASEB Journal. 2008;22(9):3287–97. 10.1096/fj.08-106831 18556463

[33] Modur V, Zimmerman GA, Prescott SM, McIntyre TM. Endothelial Cell Inflammatory Responses to Tumor Necrosis Factor α Ceramide-dependent and-independent Mitogen-activated Protein Kinase Cascades. Journal of Biological Chemistry. 1996;271(22):13094–102. 8662702 10.1074/jbc.271.22.13094

[34] Khalili JS, Liu S, Rodríguez-Cruz TG, Whittington M, Wardell S, Liu C, et al Oncogenic BRAF (V600E) promotes stromal cell-mediated immunosuppression via induction of interleukin-1 in melanoma. Clinical Cancer Research. 2012;18(19):5329–40. 10.1158/1078-0432.CCR-12-1632 22850568 PMC3463754

[35] Elkabets M, Ribeiro VS, Dinarello CA, Ostrand‐Rosenberg S, Di Santo JP, Apte RN, et al IL‐1osenberg S, D novel myeloid‐derived suppressor cell subset that impairs NK cell development and function. European journal of immunology. 2010;40(12):3347–57. 10.1002/eji.201041037 21110318 PMC3373225

[36] de Ruiter EJ, Ooft ML, Devriese LA, Willems SM. The prognostic role of tumor infiltrating T-lymphocytes in squamous cell carcinoma of the head and neck: A systematic review and meta-analysis. Oncoimmunology. 2017;6(11):e1356148 10.1080/2162402X.2017.1356148 29147608 PMC5674970

[37] Mascia F, Mariani V, Girolomoni G, Pastore S. Blockade of the EGF receptor induces a deranged chemokine expression in keratinocytes leading to enhanced skin inflammation. The American journal of pathology. 2003;163(1):303–12. 10.1016/S0002-9440(10)63654-1 12819035 PMC1868171

[38] Srivastava RM, Trivedi S, Concha-Benavente F, Hyun-Bae J, Wang L, Seethala RR, et al STAT1-induced HLA class I upregulation enhances immunogenicity and clinical response to anti-EGFR mAb cetuximab therapy in HNC patients. Cancer immunology research. 2015;3(8):936–45. 10.1158/2326-6066.CIR-15-0053 25972070 PMC4526378

[39] Concha-Benavente F, Srivastava RM, Ferrone S, Ferris RL. EGFR-mediated tumor immunoescape: the imbalance between phosphorylated STAT1 and phosphorylated STAT3. Oncoimmunology. 2013;2(12):e27215 10.4161/onci.27215 24501692 PMC3913673

[40] Tummers B, Goedemans R, Pelascini LP, Jordanova ES, Van Esch EM, Meyers C, et al The interferon-related developmental regulator 1 is used by human papillomavirus to suppress NFκB activation. Nature communications. 2015;6. 10.1038/ncomms7537 PMC4382698 26055519

[41] Pastore S, Mascia F, Mariotti F, Dattilo C, Mariani V, Girolomoni G. ERK1/2 regulates epidermal chemokine expression and skin inflammation. The Journal of Immunology. 2005;174(8):5047–56. 15814736 10.4049/jimmunol.174.8.5047

[42] Kalinowski A, Ueki I, Min-Oo G, Ballon-Landa E, Knoff D, Galen B, et al EGFR activation suppresses respiratory virus-induced IRF1-dependent CXCL10 production. American Journal of Physiology-Lung Cellular and Molecular Physiology. 2014;307(2):L186–L96. 10.1152/ajplung.00368.2013 24838750 PMC4101792

[43] Dhillon AS, von Kriegsheim A, Grindlay J, Kolch W. Phosphatase and feedback regulation of Raf-1 signaling. Cell Cycle. 2007;6(1):3–7. 10.4161/cc.6.1.3593 17218791

[44] Liu Y, Shepherd EG, Nelin LD. MAPK phosphatases-regulating the immune response. Nature Reviews Immunology. 2007;7(3):202–13. 10.1038/nri2035 17318231

[45] Valledor AF, Sánchez-Tilló E, Arpa L, Park JM, Caelles C, Lloberas J, et al Selective roles of MAPKs during the macrophage response to IFN-γ. The Journal of Immunology. 2008;180(7):4523–9. 18354174 10.4049/jimmunol.180.7.4523

